# Supplementary material for: Glycolytically impaired Drosophila glial cells fuel neural metabolism via β-oxidation
Source: Nat Commun. 2023 May 24;14:2996. doi: 10.1038/s41467-023-38813-x (PMC10209077; doi:10.1038/s41467-023-38813-x)
Supplement: Supplementary file 1 — Supplementary Information [file 41467_2023_38813_MOESM1_ESM.pdf]

Supplementary information

Supplementary Figure 1

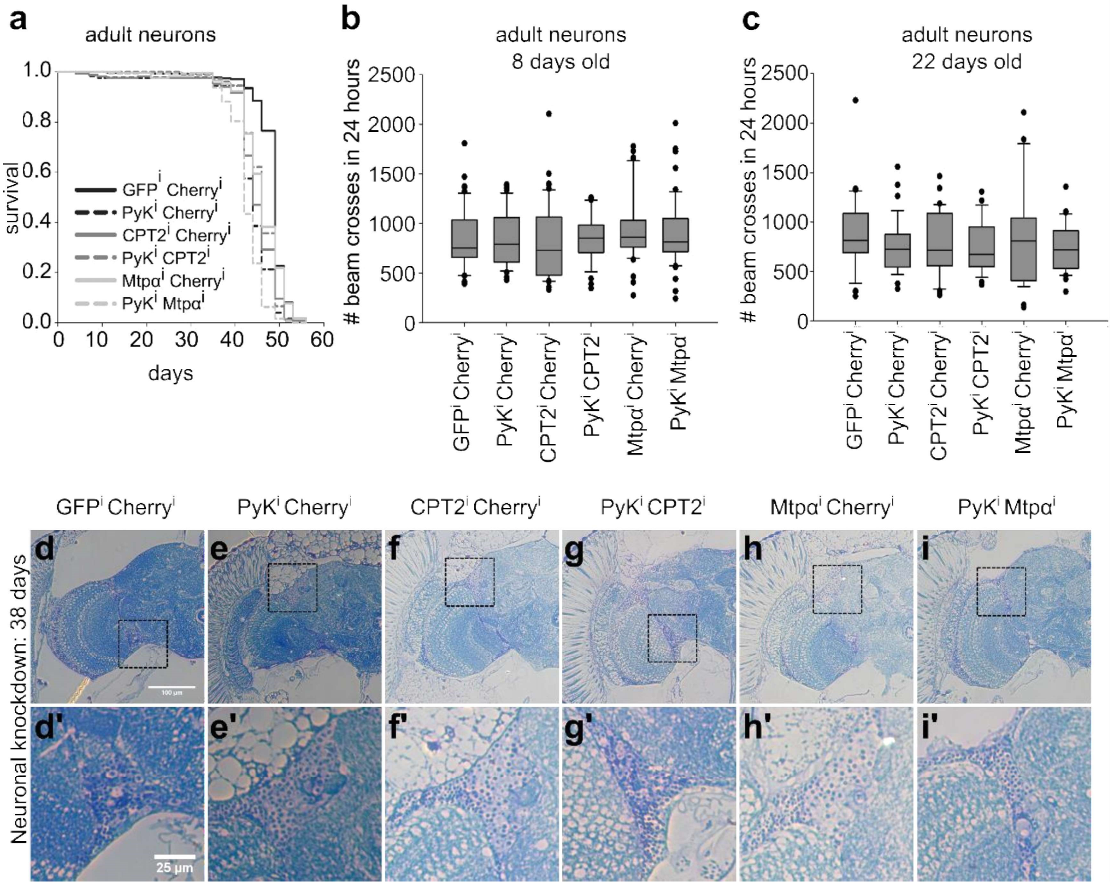

Figure S1: Neuronal knockdown of glycolysis or β-oxidation induces no phenotypic consequences a) Animals with neuronal suppression of glycolysis and/or β-oxidation show wild-typic lifespan. N=3 independent experiments, n (animals): GFP^i,Cherry^i: 182, Pyk^i,Cherry^i: 59, CPT2^i,Cherry^i: 105, Pyk^i,CPT2^i: 122, MTPα^i,Cherry^i: 159, Pyk^i,MTPα^i: 122. **b & c)** Neuronal glycolysis and/or β-oxidation knockdown animals show control locomotive activity. **b)** N=3 independent experiments, n (animals): GFP^i,Cherry^i: 50, Pyk^i,Cherry^i: 49, CPT2^i,Cherry^i: 53, Pyk^i,CPT2^i: 41, MTPα^i,Cherry^i: 47, Pyk^i,MTPα^i: 55. Two-tailed Mann-Whitney rank sum test was used for statistical analysis. No significant differences were found. Box plots: the boundary of the box indicate the 25th and 75th percentile, the line within the box marks the median. Whiskers above and below the box indicate the 90th and 10th percentiles. Dots indicate outliers. **c)** N=3 independent experiments, n (animals): GFP^i,Cherry^i: 63, Pyk^i,Cherry^i: 60, CPT2^i,Cherry^i: 54, Pyk^i,CPT2^i: 35, MTPα^i,Cherry^i: 58,

Pyk^i,$MTP\alpha^i$: 56. Two-tailed Mann-Whitney rank sum test was used for statistical analysis. No significant differences were found. Box plots: the boundary of the box indicate the 25th and 75th percentile, the line within the box marks the median. Whiskers above and below the box indicate the 90th and 10th percentiles. Dots indicate outliers. **d-i)** Histological brain semi-thin sections of neuronal glycolysis and/or β-oxidation knockdown animals show no signs of neurodegeneration (age of 38 days). All images (d-i and d'-i') are at the same scale (compare scale bar in d and d'). N=2 independent experiments, n=5 animals. Source data are provided as a Source Data file.

Supplementary Figure 2

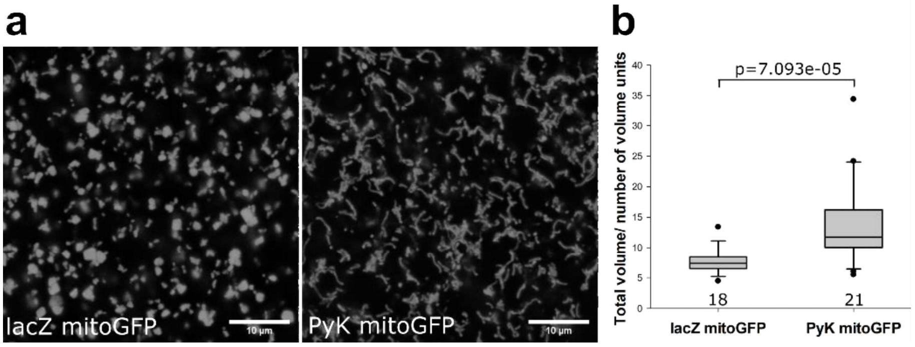

Figure S2: Switch to β-oxidation induces mitochondrial shape changes. a) Upon glial loss of glycolysis (Pyk), the mitochondria are in a more fused state then in control animals (lacZ). **b)** Total mitochondrial volume per volume units. n=18 or 21 respectively from two independent experiments (N=2). Box plots: the boundary of the box indicate the 25th and 75th percentile, the line within the box marks the median. Whiskers (error bars) above and below the box indicate the 90th and 10th percentiles. Dots indicate outliers. Wilcoxon rank sum test was used for statistical analysis. Source data are provided as a Source Data file.

Supplementary Figure 3

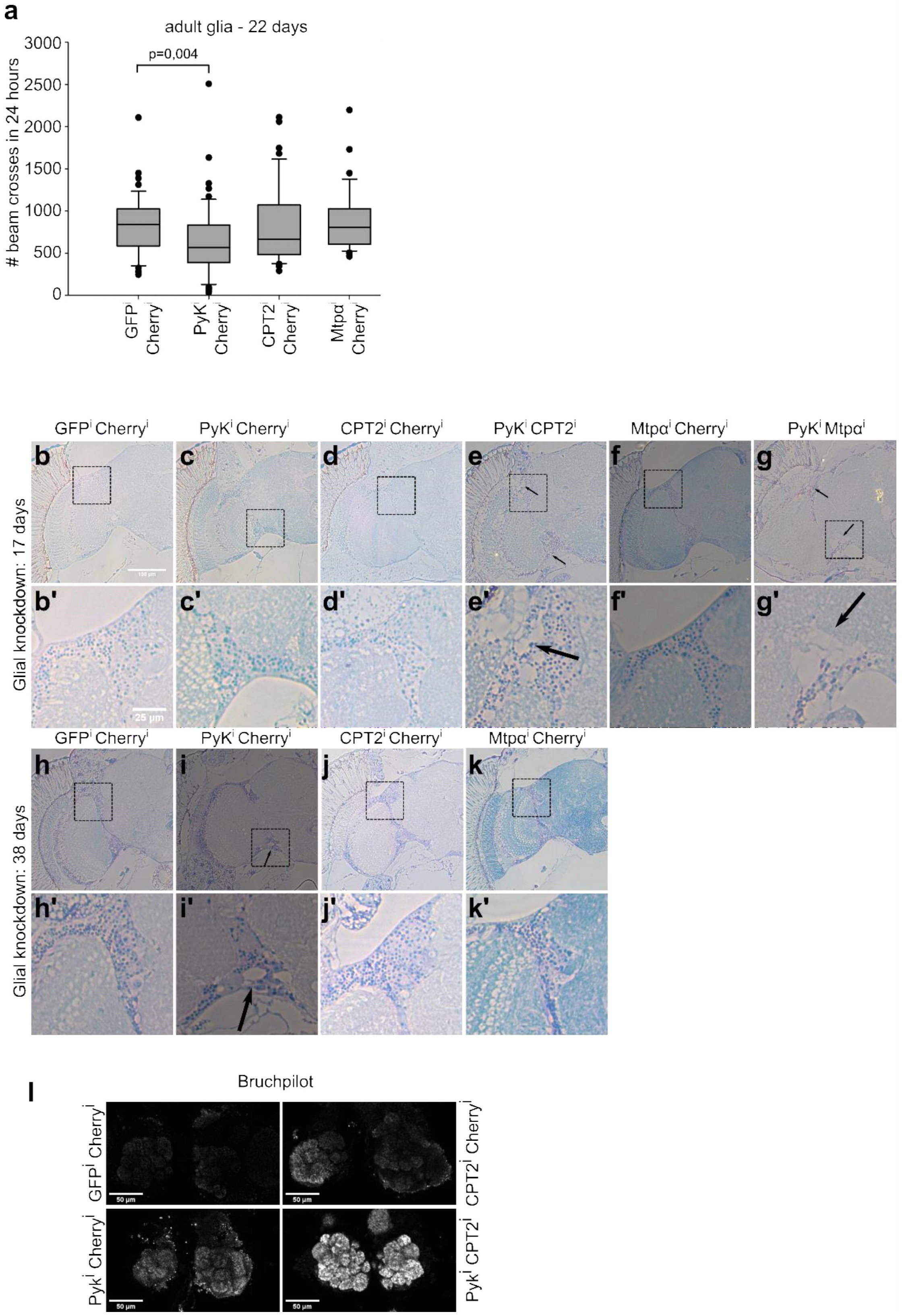

Figure S3: Loss of glial β-oxidation in addition to loss of glycolysis accelerates neurodegeneration. a) By three weeks of age locomotive activity of glial glycolysis knockdowns (Pyk, Cherry) is significantly reduced. β-oxidation knockdown animals (CPT2, Cherry and Mtpα, Cherry) show wild-typic activity. N=3 independent experiments, n (animals): GFP^i,$Cherry^i$: 51, Pyk^i,$Cherry^i$: 57, $CPT2^i$,$Cherry^i$: 61, $MTP\alpha^i$,$Cherry^i$: 66. Two-tailed Mann-Whitney rank sum test was used for statistical analysis. Box plots: the boundary of the box indicate the 25th and 75th percentile, the line within the box marks the median. Whiskers above and below the box indicate the 90th and 10th percentiles. Dots indicate outliers. **(b-k)** Semi-thin sections (one hemisphere) of adult heads. N=2 independent experiments, n=5 animals. All images are at the same scale (compare scale bar in b) (**b' – k'**) Close ups of cortex area in b-k (rectangle). All images are at the same scale (compare scale bar in b') **(b-g)** Semi-thin sections of 17-day old animals of the indicated genotypes. **(h-k)** Semi-thin sections of 38-day old animals of the indicated genotypes. Animals with simultaneous knockdown of β-oxidation and glycolysis in glia ($CPT2^i$, Pyk^i or $MTP\alpha^i$, Pyk^i) show severe signs of neurodegeneration (holes in the cortex) already at 17 days of age, while glycolysis only knockdown animals develop the neurodegenerative phenotype later (38 days). β-oxidation only knockdown or control animals do not show any signs of neurodegeneration. **l)** Brp stainings of the olfactory lobe. In glial glycolysis, β-oxidation knockdown animals Brp accumulates while control levels are found in glial glycolysis or β-oxidation single knockdown brains of animals at the age of 17 days. n=5 animals. Source data are provided as a Source Data file.

Supplementary Figure 4

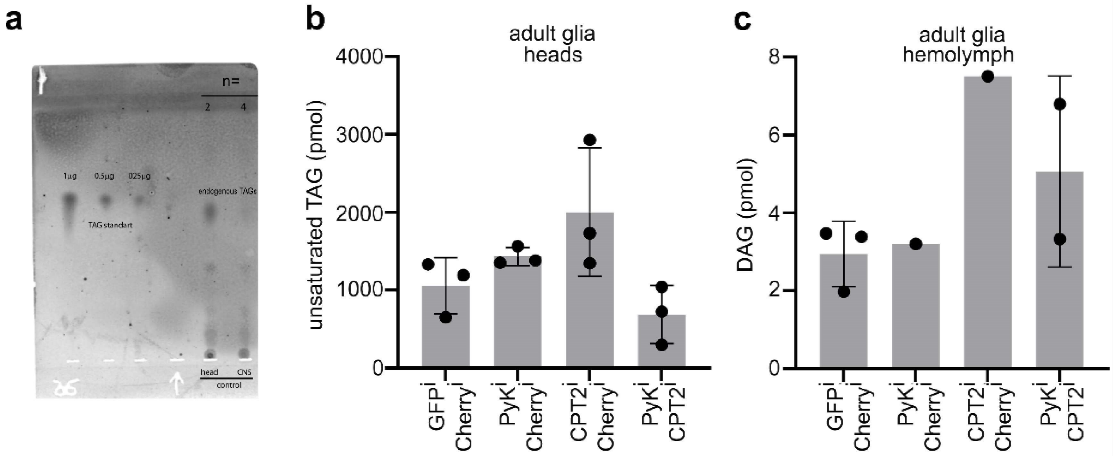

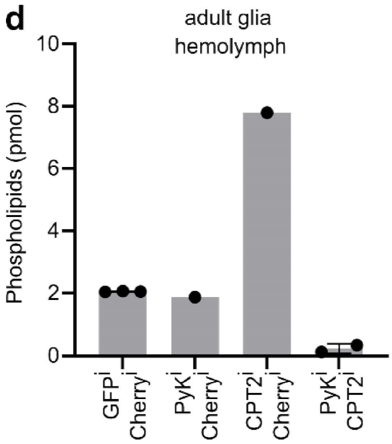

e

	GFP^i Cherry^i	PyK^i Cherry^i	CPT2^i Cherry^i	PyK^i CPT2^i
DAG/PL	1.43	1.71	0.96	21.21

Figure S4: Glial metabolic impairment induces systemic changes in lipid mobilization. a) TLC analysis shows only a faint band of TAG in the CNS samples compared to the head samples, thus TAG levels measured in head samples can be mostly attributed to the fatbody. Sample load was normalized to polar lipids. N=1 independent experiment. For the head sample two heads were used; for the brain sample four brains were used. **b)** Total amount of unsaturated TAGs in head samples. To assess significant differences between the genotypes two-tailed Mann-Whitney rank sum test was used. No significant differences are found. N =3 independent experiments with 5 animals each. Graphs: means ± standard deviation; dots are values of individual measurements. **c)** Total levels of DAGs measured in hemolymph samples. Since extracting pure hemolymph is challenging, many samples were excluded due to tissue contamination (presence of TAGs or mitochondrial PGs[34]). Nevertheless, the measurement indicates increased DAG levels in β-oxidation

knockdown as well as β-oxidation, glycolysis double knockdown animals. N= 1-3 independent experiments with 5 animals each. Graphs: means ± standard deviation; dots are values of individual measurements. **d)** Total amounts of PL detected in the hemolymph. Glial β-oxidation knockdown increases phospholipid levels. However, upon additional loss of glycolysis phospholipid levels are strongly reduced. N=1-3 independent experiments with 5 animals each. Graphs: means ± standard deviation; dots are values of individual measurements. **e)** DAG to PL ratios in the hemolymph. Source data are provided as a Source Data file.

Supplementary Figure 5

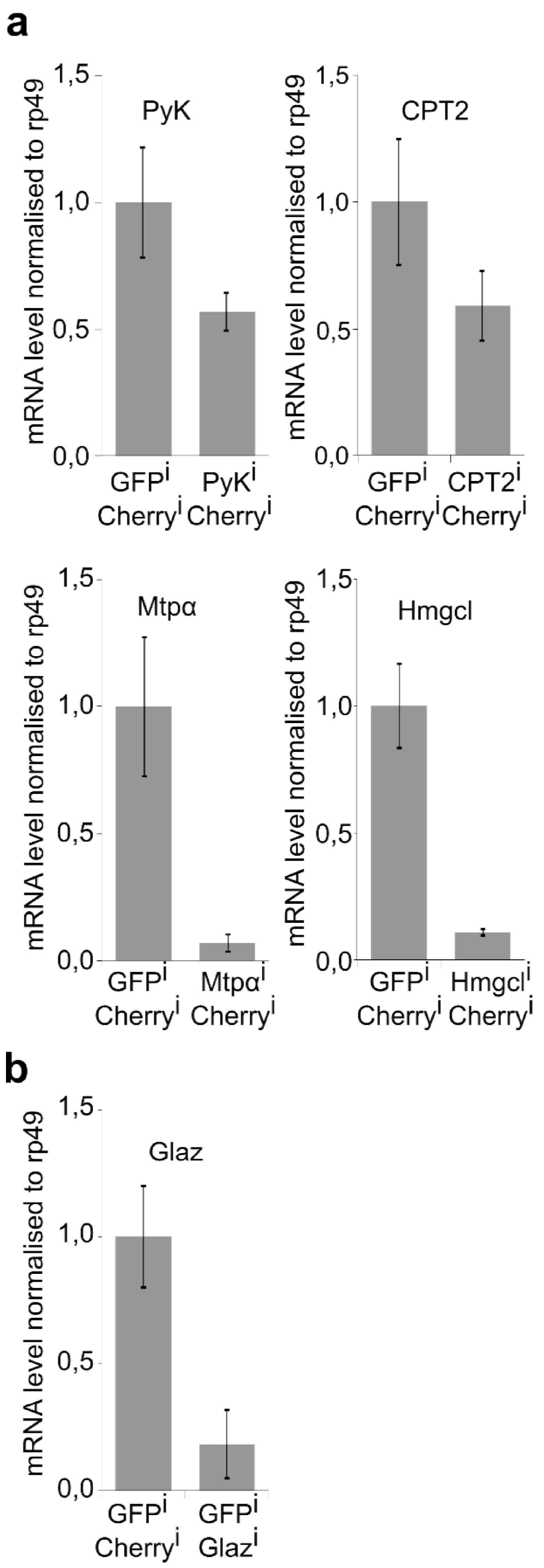

Figure S5: Analysis of dsRNA-induced knockdown efficiency. Gene expression levels were measured by quantitative RT-PCR 4 days after knockdown induction in female adult flies using the temperature-sensitive, drivers tub-Gal4, tub-Gal80^{ts} **(a)** or in female adult brains using repo-Gal4; repo-Gal4,tub-Gal80^{ts} **(b)**. Experiments have been done three times independently. Shown is data from one representative experiment each with three technical replicates. Error bars are standard deviation. Source data are provided as a Source Data file.
