# Supplementary material for: Glycolytically impaired Drosophila glial cells fuel neural metabolism via β-oxidation
Source: Nat Commun. 2023 May 24;14:2996. doi: 10.1038/s41467-023-38813-x (PMC10209077; doi:10.1038/s41467-023-38813-x)
Supplement: Supplementary file 3 — Description of Additional Supplementary Files [file 41467_2023_38813_MOESM3_ESM.pdf]

Supplementary data 1: Lifespan data
In this file the raw data for all life span experiments can be found. Each figure is represented by one work sheet in the file. All independent replicates are given separately with numbers of flies per genotype alive at each day counted.

Supplementary data 2: mass spectrometry data – CNS
Raw data of mass spectrometry analysis of brain samples. TAG: triacylglycerol, PG: phosphatidylglycerol PE: phosphatidylethanolamine PI: phosphatidylinositol, PS: phosphatidylserine

Supplementary data 3: mass spectrometry data – heads
Raw data of mass spectrometry analysis of head samples. TAG: triacylglycerol, PG: phosphatidylglycerol PE: phosphatidylethanolamine PI: phosphatidylinositol, PS: phosphatidylserine

Supplementary data 4: mass spectrometry data – hemolymph
Raw data of mass spectrometry analysis of hemolymph samples. DAG: diacylglycerol, PG: phosphatidylglycerol PE: phosphatidylethanolamine PI: phosphatidylinositol, PS: phosphatidylserine
