# Supplementary material for: Glycolytically impaired Drosophila glial cells fuel neural metabolism via β-oxidation
Source: Nat Commun. 2023 May 24;14:2996. doi: 10.1038/s41467-023-38813-x (PMC10209077; doi:10.1038/s41467-023-38813-x)
Supplement: Supplementary file 8 — Reporting Summary [file 41467_2023_38813_MOESM8_ESM.pdf]

nature portfolio

Corresponding author(s): Stefanie Schirmeier

Last updated by author(s): Apr 17, 2023

Reporting Summary

Nature Portfolio wishes to improve the reproducibility of the work that we publish. This form provides structure for consistency and transparency in reporting. For further information on Nature Portfolio policies, see our Editorial Policies and the Editorial Policy Checklist.

Statistics

For all statistical analyses, confirm that the following items are present in the figure legend, table legend, main text, or Methods section.

n/a	Confirmed	
☐	☒	The exact sample size (*n*) for each experimental group/condition, given as a discrete number and unit of measurement
☐	☒	A statement on whether measurements were taken from distinct samples or whether the same sample was measured repeatedly
☐	☒	The statistical test(s) used AND whether they are one- or two-sided<br>*Only common tests should be described solely by name; describe more complex techniques in the Methods section.*
☒	☐	A description of all covariates tested
☐	☒	A description of any assumptions or corrections, such as tests of normality and adjustment for multiple comparisons
☐	☒	A full description of the statistical parameters including central tendency (e.g. means) or other basic estimates (e.g. regression coefficient) AND variation (e.g. standard deviation) or associated estimates of uncertainty (e.g. confidence intervals)
☐	☒	For null hypothesis testing, the test statistic (e.g. *F*, *t*, *r*) with confidence intervals, effect sizes, degrees of freedom and *P* value noted<br>*Give P values as exact values whenever suitable.*
☒	☐	For Bayesian analysis, information on the choice of priors and Markov chain Monte Carlo settings
☒	☐	For hierarchical and complex designs, identification of the appropriate level for tests and full reporting of outcomes
☒	☐	Estimates of effect sizes (e.g. Cohen's *d*, Pearson's *r*), indicating how they were calculated

Our web collection on statistics for biologists contains articles on many of the points above.

Software and code

Policy information about availability of computer code

Data collection	No special software or code is central to the manuscript.
Data analysis	Lipidomics: LipidXplorer 1.2.8.1; Imaris 9.6

For manuscripts utilizing custom algorithms or software that are central to the research but not yet described in published literature, software must be made available to editors and reviewers. We strongly encourage code deposition in a community repository (e.g. GitHub). See the Nature Portfolio guidelines for submitting code & software for further information.

Data

Policy information about availability of data

All manuscripts must include a data availability statement. This statement should provide the following information, where applicable:
- Accession codes, unique identifiers, or web links for publicly available datasets
- A description of any restrictions on data availability
- For clinical datasets or third party data, please ensure that the statement adheres to our policy

The data used to generated the graphs shown in this study are provided in the Source Data file. The mass spectrometry raw data and the lifespan raw data can be found in supplementary files 1-4. The other raw datam including videos for behavioral experiments (food choice) make 160GB and thus have not been uploaded into a repository. The data will be made available upon request without undue reservation.

Human research participants

Policy information about studies involving human research participants and Sex and Gender in Research.

Reporting on sex and gender	NA
Population characteristics	NA
Recruitment	NA
Ethics oversight	NA

Note that full information on the approval of the study protocol must also be provided in the manuscript.

Field-specific reporting

Please select the one below that is the best fit for your research. If you are not sure, read the appropriate sections before making your selection.

☒ Life sciences ☐ Behavioural & social sciences ☐ Ecological, evolutionary & environmental sciences

For a reference copy of the document with all sections, see nature.com/documents/nr-reporting-summary-flat.pdf

Life sciences study design

All studies must disclose on these points even when the disclosure is negative.

Sample size	No sample-size calculation was performed. For quantitative experiments the number of animals per sample was adapted to get enough material to exceed the detection limit of the individual method. See methods section and figure legends for details. For all experiments tracking individuals sample sizes of >= 30 were chosen to account for inter-individual variation.
Data exclusions	For mass spec analysis hemolymph samples that contained cellular contamination were excluded. For the other experiments no data was excluded.
Replication	Each experiment has been performed at least twice independently, most experiments have been performed 3 times. See methods section and figure legends for details
Randomization	All animals were randomly alloted.
Blinding	Data was analyzed double blind for semi-thin sections and behavioral assays. For all quantitative analyses unbiased software-based analysis was performed.

Reporting for specific materials, systems and methods

We require information from authors about some types of materials, experimental systems and methods used in many studies. Here, indicate whether each material, system or method listed is relevant to your study. If you are not sure if a list item applies to your research, read the appropriate section before selecting a response.

Materials & experimental systems

n/a	Involved in the study	
☐	☒	Antibodies
☒	☐	Eukaryotic cell lines
☒	☐	Palaeontology and archaeology
☐	☒	Animals and other organisms
☒	☐	Clinical data
☒	☐	Dual use research of concern

Methods

n/a	Involved in the study	
☒	☐	ChIP-seq
☒	☐	Flow cytometry
☒	☐	MRI-based neuroimaging

Antibodies

Antibodies used	anti-Brp (DHB clone NC82) ; anti-Lpp - gift from Eaton lab (see reference), not commercially available.
Validation	For validation see: anti-Brp: Bruchpilot, a protein with homology to ELKS/CAST, is required for structural integrity and function of synaptic active zones in Drosophila. Buchner E Neuron 49.6 (2006 Mar 16): 833-44.

	anti-Lpp: Panáková, D., Sprong, H., Marois, E., Thiele, C., & Eaton, S. (2005). Lipoprotein particles are required for Hedgehog and Wingless signalling. Nature, 435(7038), 58–65. http://eutils.ncbi.nlm.nih.gov/entrez/eutils/elink.fcgi?dbfrom=pubmed&id=15875013&retmode=ref&cmd=prlinks

Animals and other research organisms

Policy information about studies involving animals; ARRIVE guidelines recommended for reporting animal research, and Sex and Gender in Research

Laboratory animals	Species: Drosophila melanogaster<br>Strains: Flies were maintained at room temperature on standard food. Fly crosses were kept at 18°C for 4 weeks on standard food. After hatching, mated female progeny of the correct genotype were transferred to 29 °C and flipped onto new food every second day.<br>repoGal80 (42), tubGal80ts (43), elavGal4 (44), repoGal4 (45,46), lacZi (47); tub-Gal4, tub-Gal80ts stock was a kind gift from Aurelio Teleman. Flies obtained from Bloomington Drosophila stock center: elavGal4 (BL8765, BL8760), tubGal80ts (BL7017), UAS-mito-GFP (BL8443), Cherryi (BL35785), GFPi (BL9331), CPT2HMJ23935 (BL62455), Acat1HMC03340(BL51785), HmgclHMC03435 (BL5186) ; Flies obtained from VDRC : PyKGD12123, MTPαGD1129, GlazGD4806<br>Sex: females<br>Age: adult flies, age specified in the figure legend or material and methods
Wild animals	This study did not involve wild animals.
Reporting on sex	females
Field-collected samples	This study did not involve samples collected from the field.
Ethics oversight	NA

Note that full information on the approval of the study protocol must also be provided in the manuscript.
